# The use of average Pavlov ratio to predict the risk of post operative upper limb palsy after posterior cervical decompression

**DOI:** 10.1186/1749-799X-4-24

**Published:** 2009-07-07

**Authors:** Koon-Man Sieh, Siu-Man Leung, Judy Suk Yee Lam, Kai Yin Cheung, Kwai Yau Fung

**Affiliations:** 1 Department of Orthopaedics and Traumatology, Alice Ho Mui Ling Nethersole Hospital, Tai Po, NT, Hong Kong SAR, PR China; 2 Department of Diagnostic Radiology and organ Imaging, The Chinese University of Hong Kong, Prince of Wales Hospital, Shatin, NT, Hong Kong SAR, PR China

## Abstract

**Study Design:**

A retrospective study was conducted to study the post operative upper limb palsy after laminoplasty for cervical myelopathy.

**Objective:**

To identify a reliable and simple preoperative radiological parameter in predicting the risk of post operative upper limb palsy.

**Background:**

Post operative upper limb palsy is one of the causes of patient dissatisfaction after surgery. There had been no simple, standard preoperative radiological parameters reliably predict the occurrence of this problem.

**Materials and methods:**

Seventy-four patients received posterior cervical decompression from 1998 to 2008. Medical record and preoperative radiological information were evaluated. Clinical presentations of the palsy were described. The relationship between the occurrence of palsy and different preoperative radiological information is analyzed.

**Results:**

Eighteen patients (24.3%) presented with post operative upper limb palsy. Majority of patients presented with dysesthesia (17/18) and with deficit of the C5 segment (17/18). Ten patients presented with pure dysesthesia and 8 patients presented with mixed motor-sensory deficit and dysesthesia. Multilevel involvement was exclusively presented in patients with motor weakness. A longer duration of symptom (16.7 Vs 57.2 days) was noticed in patients in the motor deficit group. Average Pavlov ratio less then 0.65 (*P *= 0.027, Odds Ratio = 3.68) and compression at the C3/4 in preoperative MRI image (*P *= 0.025, Odds Ratio = 6) were significant risk factors for development of this problem.

**Conclusion:**

Post operative upper limb palsy is not uncommon and thorough preoperative explanation is important. There is a spectrum of clinical presentation and patients with multi-level involvement and motor deficit are associated with poorer prognosis. Average Pavlov ratio < 0.65 and compression at C3/4 segment on preoperative MRI image are simple and reliable preoperative predictor for the development of this problem.

## Introduction

Cervical myeloradiculopathy caused by compression of the cervical cord by various pathologies remains one of the major disease entities of the cervical spine. Laminoplasty is simple, safe and effective in the treatment of cervical myeloradiculopathy. This technique gained widespread acceptance and popularity since the development of 'expansive open-door laminoplasty' by Hirabayashi in 1977 [1]. This has formed the basis for the development of various technique modifications.

Development of neurological deterioration after cervical operation is a major clinical problem. Post operative upper limb palsy, predominantly of the C5 segment, after cervical laminoplasty has become one of the most notorious complications affecting patients' post operative satisfaction because of the disabling symptom of paralysis and pain [2-14]. There is strong evidence on the association between post operative upper limb palsy and laminoplasty. The reported incidence of post operative upper limb palsy ranged from 0–30% [15]. There has been great disparity in the incidence and definition of this complication. Although the deficit is usually transient [1-4,9,11], long recovery time and persistent neurological symptoms had been reported [5-8,10,13]. Moreover, there has not been simple, standard preoperative radiological parameter reliably predicting the occurrence of this complication so far. The objective of the current study is to describe the clinical feature and identify a preoperative predictor for the development of post operative upper limb palsy.

## Materials and methods

A retrospective study of 74 patients undergoing posterior decompression for cervical myeloradiculopathy from 1998 to 2008 in Alice Ho Mui Ling Nethersole Hospital was conducted. There were 48 men and 26 women with mean age of 60.9 (23 to 89). The cause of cervical myeloradiculopathy included cervical spondylotic myelopathy (n = 52), ossification of posterior longitudinal ligament (n = 16), and cervical disc protrusion in developmental cervical stenosis (n = 6). Expansive open-door laminoplasty was performed and 6 patients received additional posterior instrumented fusion for concomitant instability. Medical records were reviewed by the first author (SKM). Table 1 shows their demographic characteristics and the type and level of decompression.

**Table 1:** Demographic and other characteristics of the patients

	Patient without palsy (n = 56)	Patient with Palsy (n = 18)
Sex (M/F)	36/20	12/6

Mean age at surgery (years)	60.63	61.61

Duration of symptom before operation (months)	12.42	15.06

Disease etiology (%)		
- CSM	40 (71.4)	12 (66.7)
- OPLL	11 (19.6)	5 (27.8)
- PID	5 (8.9)	1 (5.6)

Type of operation (%)		
- Laminoplasty	53 (94.6)	15 (83.3)
- Posterior decompression with internal fixation	3 (5.4)	3 (16.7)

Extent of decompression (%)		
- C2–C7	1 (1.8)	0 (0)
- C3–C7	25 (44.6)	9 (50)
- C3–C6	21 (37.5)	8 (44.4)
- C3–C5	4 (7.1)	0 (0)
- C4–C7	3 (5.4)	0 (0)
- C4–C6	2 (3.6)	1 (5.6)

Mean Preoperative JOA score	10.55	11.22

Mean Postoperative JOA score	13.66	14.22

**Recovery rate (%)**	**52.37**	**48.34**

CSM = cervical spondylotic myelopathy; OPLL = ossification of the posterior longitudinal ligament; PID = Protrusion of cervical disc in developmental cervical stenosis

*All statistically not significant

Postoperative upper limb palsy was defined as having deterioration of motor function by at least one level in standard manual muscle testing (MMT) and/or new sensory disturbance and dysesthesia with dermatomal distribution after the operation. The level of neurologic involvement was determined by the sensory dermatomal distribution and myotome involvement as follows: deltoid and biceps brachii – C5 segment, wrist extensors – C6 segment, triceps – C7 segment, wrist flexors and grip power – C8 segment, intrinsic muscles – T1 segment.

Severity of clinical symptom was described using an evaluation scores established by the Japanese Orthopaedic Association (JOA Score, Table 2). The total preoperative and postoperative JOA scores and the recovery rate, by Hirabayashi method were also calculated.

**Table 2:** Japanese Orthopaedic Association Score

**JOA SCORE**	
**I. Motor function of the upper extremity**	0. Impossible to eat with chopsticks or spoon
	1. Possible to ear with spoon, but not with chopsticks
	2. Possible to eat with chopsticks, but inadequate
	3. Possible to eat with chopsticks, but awkward
	4. Normal

**II. Motor function of the lower extremity**	0. Impossible to walk
	1. Needs cane or aid on flat ground
	2. Needs cane or aid only on stairs
	3. Possible to walk without cane or aid but slowly
	4. Normal

**III. Sensory function**	A. Upper extremity
	0. Apparent sensory loss
	1. Minimal sensory loss
	2. Normal
	B. Lower extremity (same as A)
	Trunk (same as A)

**IV. Bladder function**	0. Complete retention
	1. Severe disturbance (sense of retention, dribbling, incomplete continence)
	2. Mild disturbance (urinary frequency, urinary hesitancy)
	3. Normal



**Figure 1:**
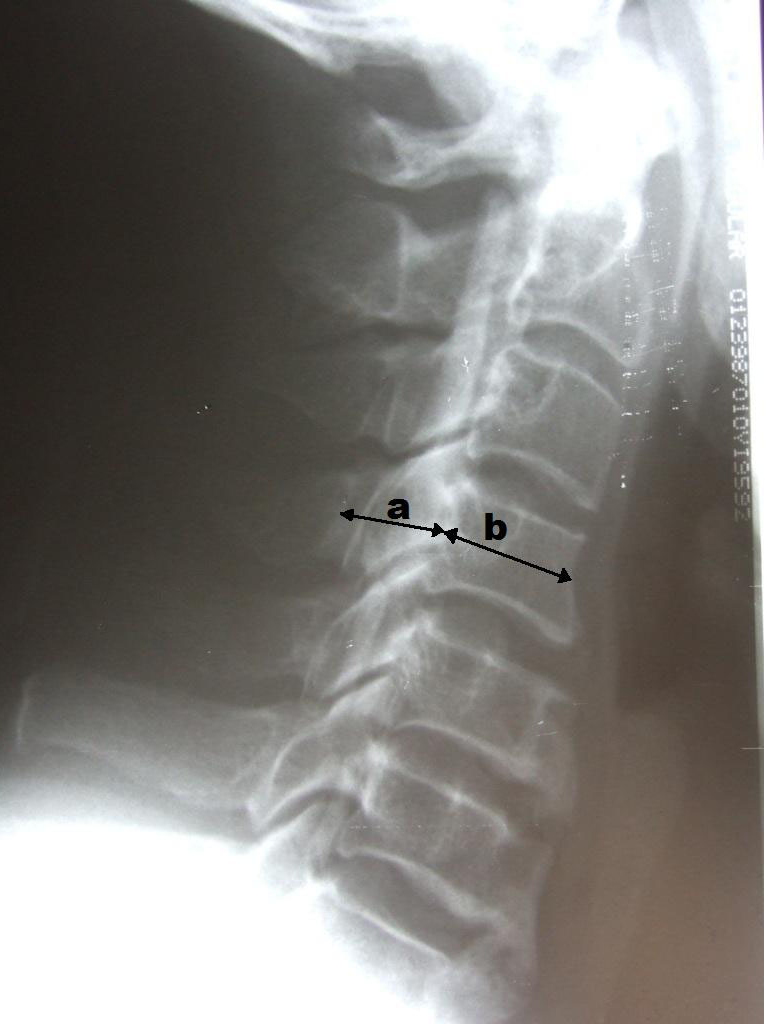
**The sagittal diameter of the spinal canal (a) is measured from the posterior point of the corresponding spinal laminar line**. The sagittal diameter of the vertebral body (b) is measured at the midpoint, from the anterior surface to the posterior surface. The spinal canal/vertebral body ratio is determined with the formula a/b as Pavlov ratio.

**Figure 2:**
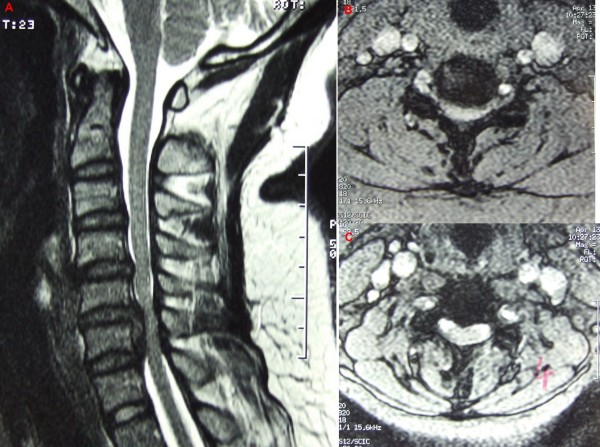
**Preoperative MRI image showing compression of the cervical cord at C5/6**. A, sagittal T2-weighted MRI image. B, corresponding axial T2-weighted MRI image showing deformation of the C5/6 by the compression. C, no deformation of the cord at C4/5.

**Figure 3:**
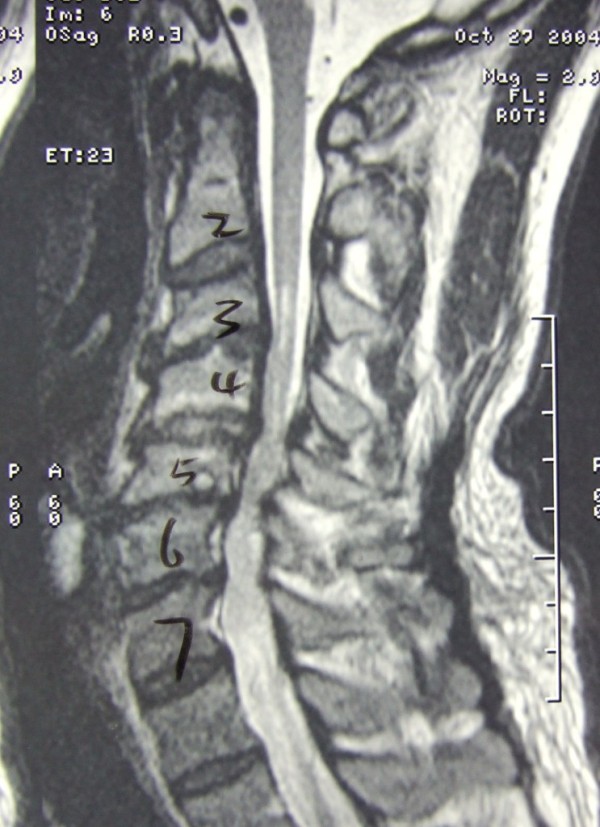
**High-signal intensity area in the C3/4 segment in T2-weighted MRI image**.

Radiologic parameters, including developmental sagittal canal diameter and vertebral body diameter from C3 to C6 were measured using a digital calliper on standard lateral cervical radiographs. The Pavlov ratio at each level and the average Pavlov ratio, calculated by averaging the Pavlov ratio at C3 to C6 level, were calculated for each patient [16-18]. (Figure 1) The alignment of the cervical spine was classified into lordosis, straight alignment, sigmoid alignment and kyphosis in accordance with the criteria of Toyama [19]. MRI was performed in every patient before the operation. Compression of cervical cord was defined as any deformation of the cervical cord shown in axial and sagittal scan of the MRI (Figure 2). The level of compression and the multiplicity of compression were also evaluated. The presence and location of high-signal intensity area (HIA) in the spinal cord on T2-weighed MRI image were recorded (Figure 3). These radiologic parameters were investigated by SKM before evaluation of the clinical notes (to minimized observer bias), and independently by an orthopaedic specialist (LSM), and a diagnostic radiologist (LJS). The mean of the three measurements was taken as final measurement to minimal inter-observer error.

### Statistical analysis

The differences in demographic characteristics and radiologic parameters, extension of decompression and degree of recovery between those with and without postoperative upper limb palsy were tested by t-test and χ^2 ^test as appropriate. Univariated analyses were performed to estimate the odds ratios of various radiologic parameters, extension of decompression and various risk factors for development of postoperative upper limb palsy. Statistical significance was defined by a *p*-value of less than 0.05. All statistical analyses were performed using by SPSS version 16.

## Results

### Post operative upper limb palsy

Eighteen patients (24.3%) developed post operative upper limb palsy between 1 and 7 days after surgery (mean 2.6 days). There was no report on the deterioration of neurological status immediately after the operation. There were no significant difference in gender, age at surgery, etiology, duration of symptom before surgery, level of decompression, pre- and postoperative JOA scores and recovery rate between patients with and without postoperative upper limb palsy (Table 1).

**Figure 4:**
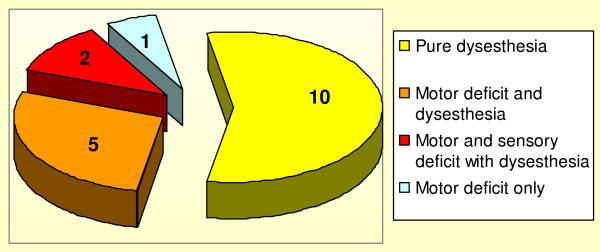
**Distribution of presentation in patients with post operative upper limb palsy**.

Ten patients presented with pure dysesthesia over the C5 dermatome and eight patients presented with motor weakness mixed with sensory deficit and dysesthesia. (Figure 4) All except one patient had C5 level involvement. The level of cervical segment involvement was showed in table 3. Multi-level involvement occurred exclusively in patients with motor weakness. Majority of these patients (7/8) presented with mixed dysesthesia, motor and sensory deficit. Twelve patients presented with unilateral symptom. Six patients presented with bilateral symptoms.

**Table 3:** Summary of patients with post operative upper limb palsy

Case no.	Age (yr)/Sex	Etiology	Presentation			Onset (days)	Duration of recovery (days)	Laterality	Level of involvement	HIA on preop. MRI
								
			Dysesthesia	Sensory deficit	Motor deficit (MMT)					
1	72/M	CSM	Yes	No	No	5	2	Left	C5	-

2	80/M	CSM	Yes	No	Yes (4 to -3)	7	42	Left	C5	C3/4, C6/7

3	71/F	CSM	Yes	No	Yes (5 to 4)	2	42	Bilateral	C5, C8	C5

4	54/F	CSM	Yes	No	No	1	8	Left	C5	C6/7

5	50/M	OPLL	Yes	Yes	Yes (5 to 3)	1	31	Right	C6, C7	C3/4

6	74/M	CSM	Yes	No	No	3	95	Right	C5	C3/4, C5/6

7	51/M	CSM	Yes	No	Yes (5 to 4)	4	5	Right	C5–7	C4/5

8	50/M	PID	Yes	No	No	3	1	Right	C5	C3–5

9	54/M	CSM	Yes	No	No	6	1	Right	C5	-

10	78/M	CSM	Yes	No	Yes (4 to -3)	1	21	Bilateral	C5–6	C5/6

11	49/F	OPLL	Yes	No	No	2	2	Left	C5	-

12	68/F	OPLL	Yes	Yes	Yes (5-4)	1	182	Bilateral	C5, C8, T1	-

13	61/F	OPLL	Yes	No	No	1	15	Left	C5	C4/5

14	49/M	CSM	Yes	No	No	1	27	Bilateral	C5	-

15	59/F	OPLL	Yes	No	No	1	11	Bilateral	C5	-

16	84/M	CSM	No	No	Yes (4-3)	4	15	Left	C5–7	C3/4, C6/7

17	60/M	CSM	Yes	No	No	2	5	Right	C5	C4/5, C5–6

18	45/M	CSM	Yes	No	Yes (5-0)	1	120	Bilateral	C5-T1	C3/4, C6/7

CSM = cervical spondylotic myelopathy; OPLL = ossification of the posterior longitudinal ligament; PID = Protrusion of cervical disc in developmental cervical stenosis; MMT = Manual muscle testing; HIA = T2 high-signal intensity area in the spinal cord;

All except one patient recovered completely from their symptom, with an average of 33.1 days (1–182 days). Twelve of the 18 patients required simple analgesic and six patients required anxiolytic and gabapentin for symptomatic relief. One patient presented with bilateral shoulder pain on the first post operative day followed by progressive weakness of both upper limb. Improvement was slow and functional recovery (MMT > 3/5) was not achieved in the latest follow-up on the 4^th ^post operative month. Those having motor weakness was older (66 Vs 58 years) and suffering from longer duration of symptom (5–182 days, mean = 57.3 days) than patient with pure dysesthesia (1–95 days, mean = 16.7 days). The difference was short of statistical significance (*p *= 0.082). (Table 4)

**Table 4:** Characteristic between patients with and without motor weakness

	Pure dysesthesia (n = 10)	Dysesthesia with motor deficit (n = 8)	*P*
Mean age (years)	58.2	65.9	0.199

Mean recovery time (days)	16.7	57.3	0.082

Sex (M/F)	6:4	6:2	0.502

HIA in T2-weighted MRI (%)	5 (50)	7 (87.5)	0.152

### Radiological data

**Figure 5:**
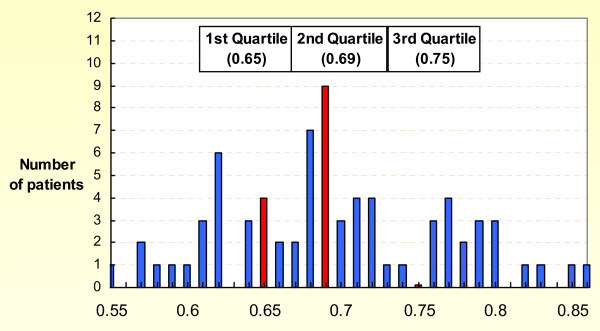
**Distribution of Average Pavlov ratio for all patients and the Quartile values**.

The mean Pavlov ratio at each level and the Average Pavlov ratio were smaller in patient with post operative upper limb palsy but statistical significance was not able to demonstrate (Table 5). Figure 5 shows the distribution of Average Pavlov ratio and the quartile value of our patients. Table 6 shows the results of the univariate analyses. Patients having severe cervical canal stenosis, defined as an Average Pavlov ratio of less than 1^st ^quartile, (0.65) (OR 3.38, *p *= 0.027) were significantly more likely to develop postoperative upper limb palsy.

**Table 5:** Pavlov ratios of patients with and without postoperative upper limb palsy

	Patient without palsy (n = 56)	Patient with palsy (n = 18)	*P*
Pavlov ratio (mean)			
C3	0.7125	0.6916	0.363
C4	0.6870	0.6701	0.495
C5	0.6836	0.6805	0.890
C6	0.7198	0.6783	0.058
Average	0.7017	0.6804	0.271

**Table 6:** Odds ratio of potential risk factors for development of postoperative upper limb palsy

	Patient without palsy (n = 56)	Patient with palsy (n = 18)	OR	*p*
Average Pavlov ratio < 0.65	10 (17.9)	8 (44.4)	3.68	0.027

Level of compression in Preoperative MRI (%)				

C2/3 (%)	3 (5.4)	1 (5.6)	1.039	0.974

C3/4 (%)	32 (57.1)	16 (88.9)	6	0.025

C4/5 (%)	40 (71.4)	16 (88.9)	3.2	0.149

C5/6 (%)	42 (75.0)	10 (55.6)	0.417	0.122

C6/7 (%)	25 (44.6)	10 (55.6)	1.55	0.421

C7/T1 (%)	1 (1.8)	0 (0)		

3 or more compression levels in MRI (%)	27 (48.2)	13 (72.2)	2.79	0.082

HIA in T2 weighted MRI image (%)	44 (78.6)	11 (61.1)	0.955	0.943

Compression of cervical cord in preoperative MRI was most commonly occurring at the mid-cervical level (Table 6). C4/5 level in 56 patients (71.8%), followed by C5/6 in 52 patients (66.7%) and C3/4 in 48 patients (64.9%). However, compression at C3/4 level is showed to associate with higher risk of occurrence of palsy (OR 6, *p *= 0.025). Multiplicity of compression, defined by 3 or more compression from preoperative MRI, showed higher rate of development of post operative upper limb palsy (73.2% Vs 48.2%) with marginal statistical significance (*p *= 0.082). The association between palsy and preoperative alignment, intramedullary high-signal intensity area (HIA) on preoperative T2-weighted MRI were not significant.

## Discussion

Post operative upper limb palsy after cervical laminoplasty posterior has raised substantial concern in the past 20 years but controversies still remain in the nomenclature, pathophysiology and defining the risk factors for the development of this significant complication. 'Post operative C5 palsy', defined by unilateral paralysis of the deltoid and biceps muscles without sensory disturbance [2,3,20] is one of the commonly used definitions among surgeons. At the same time, there are considerable concerns about the multilevel motor paralysis after the surgery [4-10,21,22], which led to the evolution of different nomenclatures like 'Segmental motor paralysis' [4-6], 'Post operative muscle weakness of the upper extremities' [8], 'Post operative motor paralysis of the upper limb' [10] and 'upper extremity palsy' [7,22]. Moreover, some authors who also include pain and sensory disturbance in describing this problem [5,7,9,11,13,14]. In this study, we defined postoperative upper limb palsy as having deterioration of motor function by at least one level in standard manual muscle testing (MMT) and/or new sensory disturbance and dysesthesia with dermatomal distribution after the operation.

Narrow spinal canal is associated with higher risk for the development of cervical myelopathy [16-18]. Edwards et al. defined narrow spinal canal by direct measurement of the midcervical diameter from standard lateral cervical radiograph. Measurement less then 13 mm is prone to development cervical myelopathy [18]. The use of Pavlov ratio eliminated the discrepancy of magnification and had been generally accepted an essential radiological parameter in management of cervical myeloradiculopathy. Pavlov ratio less then 0.82 are considered stenotic [17] and are associated with a higher risk of cervical myelopathy. Yue et al. echoed the work of Pavlov and concluded that Average Pavlov ratio might be a useful predictor to cervical myelopathy [17]. However, the association of narrow spinal canal with the risk of post operative upper limb palsy has not been clearly established.

Recognizing the substantial proportion of multi-level involvement in our patient with the post operative upper limb palsy, we hypothesize that pathological insults to the cervical cord adopted a similar 'multi-level' fashion. We also hypothesize that patients with developmental cervical stenosis, implied by a small Average Pavlov ratio, will have higher risk for developing postoperative palsy. A developmentally narrowed spinal canal made cord compression more likely. On the other hand, there may be inherent factors associating with the narrowing which render the cord more susceptible to pathological insults, e.g. peculiar blood supply, or as a result of reperfusion after decompression of multi-level compression [15].

Since our patients were all suffering from symptomatic cervical myeloradiculopathy, the distribution of Average Pavlov ratio is expected to be skewed instead of normal, which justified the transformation of the Average Pavlov ratio into a categorical variable. We used the 1^st ^quartile as the cut off for defining extremely narrow spinal canal, for subsequent analyses (Figure 5). We are able to show a significantly higher risk of developing postoperative palsy in patients having an Average Pavlov ratio of less then 0.65 with an odds ratio of 3.68. Moreover, there was a higher rate of post operative upper limb palsy in patient with 3 or more compression on preoperative MRI with marginal statistical significance. These results support our assumption that an extremely narrow cervical spinal canal is prone to development of post operative upper limb palsy.

Majority of our patients present with dysesthesia (17/18) and had neurologic deficit involving the C5 segment (17/18). We showed compression at the C3/4 level is strongly associated with the development of palsy. Since anatomical study showed C3/4 level corresponds to the C5 cord segment [23], it is reasonable to assume compression of C5 segment is a strong contributing factor in the development of post operative upper limb palsy and this also explained the predilection of C5 neurologic dysfunction.

There is no significant difference in JOA score and recovery rate between patients with and without the palsy in this study. Since the effect of post operative upper limb palsy is transient, most of the patients had already recovered during the follow up review in this retrospective study. Further this may be the limitation of the JOA score in reflecting the functional disturbance attributed by the post operative upper limb palsy.

Although many researchers has suggested different preoperative and intraoperative monitor in detecting the occurrence and evaluating the risk of post operative upper limb palsy, they usually involved specially trained personnel and sophisticated equipment [2,11,20-22]. In our pilot study using measuring ruler in measuring the Average Pavlov ratio, same conclusion (0.65) can be reached. This suggested simple measuring technique is also applicable when using Average Pavlov ratio to predict the risk of occurrence of palsy. Our study is able to demonstrate these two preoperative radiological parameters are simple, reliable in predicting this significant post operative complication of posterior cervical decompression.

Finally, this is the drawback of the current study of small population size. The dose-effect of spinal canal narrowing to the development of post operative upper limb palsy was not able to demonstrate and causing statistical insignificant or marginal significant in variable parameters.

## Conclusion

Post operative upper limb palsy is a significant post operative complication of cervical posterior decompression that thorough preoperative explanation is important. Spectrum of presentation from pure dysesthesia to multi-level, motor sensory dysfunction is demonstrated. Patient presented with multi-level involvement and motor dysfunction is associated with longer recovery period. Average Pavlov Ratio of less then 0.65 and cervical cord compression of C3/4 level in preoperative MRI could be simple and reliable predictors for the development of post operative upper limb palsy

## Competing interests

The authors declare that they have no competing interests.

## Authors' contributions

All authors had substantial contributions to conception and design of the study and giving final approval to the manuscript. KMS, SML and JSYL participated in the data acquisition. KMS is responsible for data interpretation and writing of the manuscript. All authors have read and approved the final manuscript.
